# Preferential *MGMT* hypermethylation in SDH-deficient wild-type GIST

**DOI:** 10.1136/jcp-2022-208462

**Published:** 2022-10-05

**Authors:** Olivier T Giger, Rogier ten Hoopen, David Shorthouse, Shukri Abdullahi, Venkata Ramesh Bulusu, Saili Jadhav, Eamonn R Maher, Ruth T Casey

**Affiliations:** 1 Pathology, University of Cambridge, Cambridge, UK; 2 Oncology, University of Cambridge, Cambridge, UK; 3 Department of Medical Physics and Biomedical Engineering, University College London, London, UK; 4 Oncology, Addenbrooke's Hospital, Cambridge, UK; 5 Department of Medical Genetics and Cancer Research, University of Cambridge, Cambridge, UK

**Keywords:** Gastrointestinal Neoplasms, Stomach Neoplasms, Sarcoma, Neoplastic Syndromes, Hereditary, Neuroendocrine Tumors

## Abstract

**Aims:**

Wild-type gastrointestinal stromal tumours (wtGIST) are frequently caused by inherited pathogenic variants, or somatic alterations in the succinate dehydrogenase subunit genes (*SDHx*). Succinate dehydrogenase is a key enzyme in the citric acid cycle. SDH deficiency caused by *SDHx* inactivation leads to an accumulation of succinate, which inhibits DNA and histone demethylase enzymes, resulting in global hypermethylation. Epigenetic silencing of the DNA repair gene MGMT has proven utility as a positive predictor of the therapeutic efficacy of the alklyating drug temozolomide (TMZ) in tumours such as glioblastoma multiforme. The aim of this study was to examine MGMT promoter methylation status in a large cohort of GIST.

**Methods:**

MGMT methylation analysis was performed on 65 tumour samples including 47 wtGIST (33 SDH-deficient wtGIST and 11 SDH preserved wtGIST) and 21 tyrosine kinase (TK) mutant GIST.

**Results:**

*MGMT* promoter methylation was detected in 8 cases of SDH-deficient (dSDH) GIST but in none of the 14 SDH preserved wild-type GIST or 21 TK mutant GIST samples analysed. Mean MGMT methylation was significantly higher (p 0.0449) and MGMT expression significantly lower (p<0.0001) in dSDH wtGIST compared with TK mutant or SDH preserved GIST. No correlation was identified between *SDHx* subunit gene mutations or SDHC epimutation status and mean MGMT methylation levels.

**Conclusion:**

MGMT promoter hypermethylation occurs exclusively in a subset of dSDH wtGIST. Data from this study support testing of tumour MGMT promoter methylation in patients with dSDH wtGIST to identify those patients who may benefit from most from TMZ therapy.

**WHAT IS ALREADY KNOWN ON THIS TOPIC**

- Gastrointestinal stromal tumours (GIST) with mutations in the succinate dehydrogenase (SDH) protein complex are resistant to current treatment regimes in other GIST. Alkylating agents may be used for treatment of tumours which show loss of MGMT function due to DNA methylation of its promoter. The aim of this study was to assess whether MGMT promoter methylation was a common finding in SDH-deficient GIST.

**WHAT THIS STUDY ADDS**

- Our data shows that *MGMT* promoter methylation is frequent if not exclusive to SDH-deficient GIST. *The tumour MGMT* methylation status may also have a role as a prognostic biomarker and could inform on potential therapeutic options for SDH-deficient GIST.

**HOW THIS STUDY MIGHT AFFECT RESEARCH, PRACTICE OR POLICY**

- Data from this study suggest that diagnostic testing for *MGMT* promoter methylation should be considered as part of diagnostic clinical testing for SDH-deficient GIST.

## Background

Gastrointestinal stromal tumours (GISTs) are mesenchymal tumours of the gastrointestinal tract with an incidence of 15–20 per million of the population.^1 2^ Most GISTs occurring in adults are driven by activating somatic mutations in the receptor tyrosine kinase genes *KIT* 3 or *PDGFRA* 4 and mutations in these proto-oncogenes predict an excellent response to tyrosine kinase inhibitors.^5^ Wild-type GIST (wtGIST) refers to those which are negative for activating mutations in *KIT* and *PDGFRA*.^5^ These account for 15% of adult and 85% of paediatric GIST. The majority of wtGIST are caused by a loss of function in the succinate dehydrogenase (SDH) enzyme complex, most commonly caused by an inherited mutation in one of the four *SDHx* genes (*SDHA*, *SDHB*, *SDHC* and *SDHD*)^5^ or by tumour-specific *SDHC* silencing by promoter methylation.^6^ *SDHx* mutations impair SDH enzyme complex assembly at the inner mitochondrial membrane or cause a loss of the enzyme complex function. These tumours are therefore referred to as SDH deficient (dSDH). The association of *SDHx* mutations with a hereditary tumour syndrome was first described in familial phaeochromocytoma and paraganglioma (PPGL).^7^ Over the past two decades, the spectrum of tumours associated with SDH deficiency has been extended to include GIST, renal cell carcinomas (RCC) and pituitary adenomas.^8^ The SDH enzyme couples the oxidation of succinate to fumarate in the citric acid cycle and a loss of function in tumour cells leads to accumulation of succinate. Excess levels of succinate inhibit the 2-oxyglutarate dependent dioxygenase enzymes including the Jumonji C (JmjC) histone demethylase class of enzymes and the ten eleven translocase DNA demethylase enzymes.^9^ Genome-wide methylation profiling of *SDHx* mutated tumours has demonstrated DNA hypermethylation in PPGL^9^ and wtGIST.^10^ This has prompted interest in the potential therapeutic utility of precision medicine approaches targeting hypermethylated tumour suppressor genes in these tumours.

6-methylguanine-DNA methyltransferase (*MGMT*) encodes a DNA repair protein that removes alkyl groups from the guanine residue within DNA. DNA alkylation most commonly occurs at guanine residues (O6-guanine, N7-guanine) and leads to single and double-strand DNA breaks and therefore, if not repaired, to subsequent apoptotic cell death. *MGMT* expression within cancer cells allows the cell to recover from the DNA damaging effects of alkylating agents enabling the tumour to become resistant to therapeutic use of such agents. Epigenetic silencing of *MGMT* by promoter hypermethylation has been described in malignancies of the colon and rectum (39%), central nervous system (34%), head and neck (32%), lung (21%), lymphoma (25%), oesophagus (20%) and pancreas (11%).^11^ The status of *MGMT* expression has been proven to be of significant clinical benefit in the management of glioblastoma multiforme, where epigenetic silencing of *MGMT* by promoter hypermethylation informs therapeutic response to temozolomide.^12^ A correlation between germline *SDHB* status and *MGMT* promoter methylation has been demonstrated in PPGL^13^ and the authors of this study postulated that the reduced *MGMT* expression due to promoter hypermethylation was responsible for the favourable response to temozolomide (TMZ) in the cohort of patients with *SDHB* mutations. More recently, *MGMT* was found to be preferentially methylated in a small subset of SDH-deficient (dSDH) wild-type GIST compared with a larger subset of SDH preserved (pSDH) wild type GIST (6/9 (67%) dSDH GIST, vs 6/39 (15%) pSDH-preserved GISTs.^14^ Lou *et al* observed a significantly higher percentage of MGMT promoter hypermethylation in SDH deficient and epithelioid/mixed non-TK mutant GIST (4/7 and 8/44, respectively).^15^

At present, there are few effective oncological therapies to treat patients with inoperable metastatic dSDH wtGIST. The outcome of an open-label, phase 2 efficacy study of TMZ in advanced SDH-mutant/deficient wtGIST (ClinicalTrials.gov Identifier: NCT03556384) is awaited but earlier studies have suggested that *MGMT* methylation status could be used as a biomarker to identify individuals with metastatic wtGIST, who might have a favourable response to TMZ therapy.

### Study aims

The aims of this study were (1) to profile *MGMT* promoter methylation status and *MGMT* expression in a large UK cohort of wtGIST and (2) to inform the utility of *MGMT* methylation analysis as a routine clinical diagnostic test for patients with metastatic wtGIST for whom systemic therapy is being considered.

## Methods

### Clinical sample collection

Cases were ascertained from the National Paediatric and Adult wild type GIST (PAWS GIST UK) and GIST clinic at Cambridge University Hospital NHS Foundation Trust. Details of clinical phenotype, family history, histopathology and germline molecular testing results were collated from patient records.

### Study design

This was a retrospective study. wtGIST patients, for whom formalin-fixed paraffin embedded (FFPE) tumour blocks or fresh frozen (FF) tumour tissue were available, were eligible for inclusion. The SDH status for all tumours had been assessed by SDHB immunohistochemistry. A control set of *KIT*, *PDGFRA*, *NF1* and quadruple-wt GISTs (n=32) was included. The FFPE tumour blocks from the primary tumour were available for all cases and FF tissue from the primary tumour was available for four cases. Analysis was also performed for a subset of patients (n=2) for whom FFPE tumour blocks from the primary and metastatic tumour were available.

### Tissue dissection for DNA and RNA isolation

Preselected paraffin blocks containing tumour were used for molecular analysis. Histologically confirmed tumour and tumour-free tissue suitable for DNA isolation was identified by an experienced molecular histopathologist (OG). The tumour cell content in the selected areas ranged between 50% and 80%. The 6–10 µm thick FFPE sections were mounted on glass slides. Tumour and normal tissue were scraped of the slides barring a security margin between tumour and normal of 2 mm.

### Clinical germline DNA sequencing

DNA was extracted from peripheral blood samples according to standard protocols. Next-generation sequencing of a clinical gene panel including; *SDHA*, *SDHB*, *SDHC*, *SDHD*, *KIT, PDGFRA* and *NF1* was performed by the laboratory staff at Cambridge University Hospital NHS Foundation Trust or Birmingham Women’s and Children’s Hospital NHS Trust using the TrusightOne or Trusight Cancer sequencing panels (Illumina, UK). An average coverage depth of >20 fold was achieved for 98% of the regions sequenced. All detected variants were confirmed by Sanger sequencing. Whole exon deletions and duplications and large rearrangements are not detected using this method and multiple ligation probe analysis was performed for *SDHB*, *SDHC* and *SDHD*.

### DNA extraction

DNA was extracted from FFPE tissue according to standard protocols. For details, please see online supplemental data.

10.1136/jcp-2022-208462.supp1 Supplementary data

### RNA extraction from FF tissue

RNA from FF and FFPE tissue was isolated according stardard protocols. For details, please refer to online supplemental data.

### Bisulfite conversion

Bisulfite conversion was performed using the Qiagen Epitect Bisulfite kit (Cat 59104) or the Zymo Research EZ DNA Methylation kit (D5001) according to the manufacturers’ instructions. viii) Analysis of *MGMT* and *SDHC* promoter methylation.

1–25 ng bisulfite converted DNA was used for *MGMT* and *SDHC* promoter methylation analysis.

For *MGMT* 375 nM forward primer, with a 20-mer 5’ M13 overhang (TGTAAAACGACG-GCCAGTTTATAGTTTYGGATATGTTGGGATAG) and 187.5 nM of biotinylated reverse primer ((btn)-TCCCAAACACTCACCAAATC) were used. For sequencing a nested sequencing primer (GTTTTTAGAACGTTTTGYGTTT) was used.

For *SDHC* 375 nM forward primer a 20-mer 5’ M13 overhang, (TGTAAAACGACGGCCAGTTTATAGGAGAAGTTTTAGAGTTTTTTAAAGAG) and 250 nM of biotinylated reverse primer ((btn)-AAAATAACRCCAAACRACCCC) were used. For *SDHC* a nested sequencing primer (GTTATATGATATTTTTAATTT) was used. *MGMT* promoter hypermethylation was defined as a mean *MGMT* methylation and *SDHC* across CpG islands 1–8 of >10% for this study. For details,please refer to online supplemental data.

### MGMT expression analysis with quantitative RT-PCR

RNA (125–250 ng) was transcribed into cDNA with random hexamers. Relative *MGMT* expression was analysed according to^16 17^ with SYBR Green using the PowerUp SYBR Green Master Mix (Applied Biosystems, ref 01061935), with *MGMT* oligo’s; forward 5'-GCTGAATGCCTATTTCCACCA-3'/reverse 5'-CACAACCTTCAGCAGCTTCCA-3'; normalised to the average Ct value of three internal reference genes: *HPRT1*, *GUSB*, *TBP*). The delta Ct was calculated by subtracting the mean of triplicate Ct values for *MGMT* with the mean of the triplicate Ct values of all three reference genes.

### Statistical analysis

Statistical analyses were performed using GraphPath Prism V.6. Groups were compared by ANOVA (Kruskall-Wallis), assuming non-Gaussian distribution. Comparisons between *MGMT* methylation and *MGMT* expression between groups was performed using unpaired Mann-Whitney t-test assuming a non-Gaussian distribution. Analysis of *MGMT* methylation in tumours vs adjacent normal tissue was performed using a paired t-test and correlations between mean *MGMT* methylation and clinical and pathological features was performed using an unpaired t-test.

### Analysis of public data

**Figure 1:**
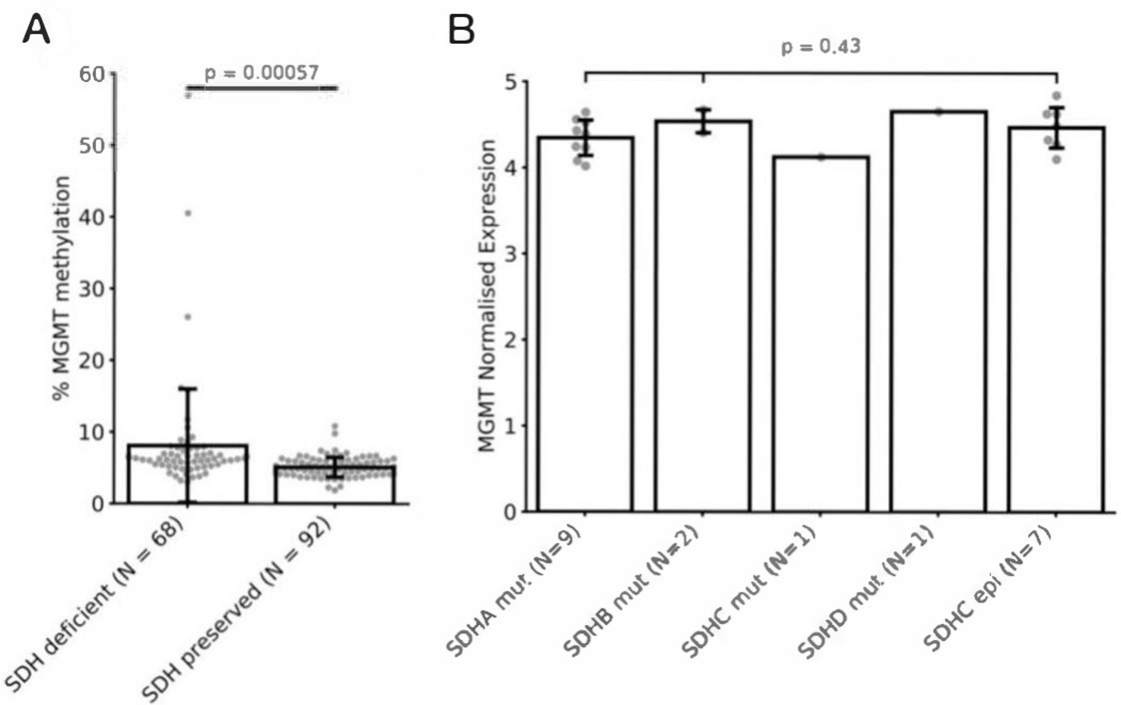
Assessment of *MGMT* promoter status in previously published GIST data. (A) *MGMT* probe methylation status for GIST patients dSDH (N=68) and pSDH (N=92) from Killian *et al*.^10^ P value represents Student’s t-test. (B) RNA normalised expression for *MGMT* in GIST patients with different mutations in SDH genes from Killian *et al*.^18^ P value represents one-way analysis of variance (ANOVA). GIST, gastrointestinal stromal tumour.

Methylation and affymetrix expression data for GISTs was downloaded from the repositories for Killian *et al* 10 and Killian *et al* 18 (GEO ids 34 387 and 56 670, respectively) (15)(10). PPGL data were downloaded from online supplemental table S2 of Hadoux *et al*.^16^ TCGA data were downloaded using Xenabrowse^19^ the ‘Pan-cancer atlas’.^20^ *MGMT* methylation for figure 1A was calculated using the average (mean) of both available probes (MGMT_P272_R, MGMT_P281_F). For PPGL data, *MGMT* promoter methylation was calculated as the average (mean) of probes found to be differently methylated and that correlate with *MGMT* expression in Hadoux *et al* (cg25946389, cg12434587, cg12981137, cg02941816).^16^ Analysis was performed using Python. P values represent Student’s t-tests, one-way ANOVA or Tukey post hoc tests performed using the SciPy^21^ (Student’s t-tests, ANOVA) or statsmodels (Tukey) libraries.

10.1136/jcp-2022-208462.supp2 Supplementary data

## Results

*MGMT* methylation analysis was performed on 68 tumour samples from 65 individual patients including 47 wtGIST (67.7%) and 21 (32.3%) TK mutant (17 *KIT* and 4 *PDGFRA*) GIST (30.9%). Complete clinical and pathological data was available for 54 patients (79.4%). The mean age in the study cohort was 46.7 years (range 13–79 years). The cohort included 32 female (49.3%) and 24 male (36.9%) patients (information on gender was not available for 9 patients (13.8%)).

Nineteen (29.2%) patients had metastatic disease and eight patients had synchronous tumours (six PPGL and two pulmonary chondromas; see table 1); (see online supplemental table S1). A germline *SDHx* mutation was identified in 17 cases of dSDH wtGIST. Tumour *SDHC* promoter methylation was identified in 12 cases of dSDH wtGIST of which nine had no identifiable germline *SDHx* mutation. The germline genetic test results were unavailable for four cases.

**Table 1:** Clinicopathological features of study cohort

Clinicopathological features	N=(%)
Age	Mean age 46.7 years (range 13–79 years)
Gender	32 female (49.3%), 24 male (36.9%), 9 unknown (13.8%)
Primary site of GIST	Gastric 45 (69.2%), small bowel 12 (18.5%), unknown 8 (12.3%)
Metastatic	Yes 19 (29.2%), no 38 (58.5%), unknown 8 (12.3%)
Synchronous tumour	Yes 8 (12.3%) (6 PPGL, 2 pulmonary chondroma) No 49 (75.4%), unknown 8 (12.3%)
Histological subtype of GIST	Epithelioid 7 (10.8%), mixed 23 (35.4%), spindle 14 (21.5%) unknown 21 (32.3%)
Proliferation index	Mean 5% range (1%–80%)

GIST, gastrointestinal stromal tumour.

### MGMT methylation and correlation with molecular status

Mean *MGMT* promoter methylation level for the 65 GIST studied varied by GIST subgroup: mean *MGMT* methylation was 2.905% (SEM=1.758) for TK mutant GIST (N=21), 3.143% (SEM=0.4041) for NF1 (N=7) vs 2.25% (SEM=0.75 SEM) for quadruple negative GIST (N=4) and 8.091% (SEM=1.786) for SDH-deficient GIST (N=33) (p=0.0449)(table 2). Overall *MGMT* promoter methylation (defined as mean methylation >10%) was identified in 8 of 65 cases (12.3%). All 8 cases were dSDH wtGIST (8/33, 24.2%). *MGMT* promoter hypermethylation was not identified in any of the 11 SDH preserved wtGIST or the 21 TK mutant GIST samples analysed. There was no difference in mean methylation comparing SDH tumours caused by *SDHC* epimutation (mean methylation 7.5566.583% (SEM=2.897207) to those with germline mutations in *SDHA* (mean methylation 4.556% (SEM=1.192) or other *SDHx* subunit gene mutations (*SDHB/C/D*) (mean methylation 8.5% (SEM 5.099), Kruskall-Wallis ANOVA p=0.9167.

**Table 2:** Molecular features of the SDH-deficient GIST cohort

Tumour ID	KIT/PDGFRA mutation status	SDH status	Germline gene mutation	SDHC epimutation	Tumour SDHC methylation	Mean MGMT methylation
G0001	WT	dSDH	No	Y	76%	2%
G0002	WT	dSDH	*SDHC c.380A>G (p. His127Arg*)	Y	46%	2%
G0003	WT	dSDH	No	N	1%	23%
G0006	WT	dSDH	*SDHA c.1765C>T (p. Arg589Trp*)	N	5%	5%
G0010	WT	dSDH	*SDHD c.296delT (p. Leu99fs*)	N	2%	44%
G0011	WT	dSDH	*SDHA c.91C>T (p. Arg31Ter*)	N	2%	3%
G0012	WT	dSDH	SDHD c.34G>A (p. Gly12Ser*)	Y	46%	4%
G0013	WT	dSDH	No germline pathogenic variant detected	Y	54%	5%
G0017	WT	dSDH	No germline pathogenic variant detected	Y	79%	3%
G0018	WT	dSDH	*SDHB c.137G>A (p. Arg46Gln*)	N	2%	2%
G0019	WT	dSDH	*SDHC c.148C>T (p. Arg50Cys*)	Y	32%	5%
G0020	WT	dSDH	*SDHC c.43C>T (p. Arg15X*)	N	2%	3%
G0021	WT	dSDH	*SDHA c.91C>T (p.Arg31Ter*)	Y	5%	8%
G0024	WT	dSDH	No germline pathogenic variant detected	N	4%	7%
G0025	WT	dSDH	SDHA c1A>C, (p.MET1?)	N	7%	3%
G0026	WT	dSDH	*SDHB c.72+1G>T*	N	4%	6%
G0027	WT	dSDH	*SDHA c.150+1G>A*	N	2%	5%
G0029	WT	dSDH	*SDHA c.91C>T (p. Arg31Ter*)	N	6%	2%
G0030	WT	dSDH	*SDHA c.91C>T (p. Arg31Ter*)	N	4%	3%
G0053	WT	dSDH	No germline pathogenic variant detected	Y	47%	4%
G0057	WT	dSDH	No germline pathogenic variant detected	N	4%	15%
G0081	WT	dSDH	*SDHB c.72+1G>T*	N	1%	2%
G0082	WT	dSDH	No germline pathogenic variant detected	Y	80%	19%
G0085	WT	dSDH	No germline pathogenic variant detected	Y	31%	3%
G0086	WT	dSDH	No germline pathogenic variant detected	N	3%	3%
G0140	WT	dSDH	NA	N	2%	23%
G0141	WT	dSDH	NA	N	2%	33%
G0142	WT	dSDH	NA	Y	77%	3%
G0143	WT	dSDH	NA	N	2%	2%
G0144	WT	dSDH	*SDHA c.1909–2A>G*	N	2%	12%
G0150	WT	dSDH	No germline pathogenic variant detected	Y	68%	3%
G0151	WT	dSDH	No germline pathogenic variant detected	Y	69%	26%
G0177	WT	dSDH	*SDHA c.91C>T (p. Arg31Ter*)	N	2%	0%

GIST, gastrointestinal stromal tumour; SDH, succinate dehydrogenase.

### MGMT expression

*MGMT* expression by qRT-PCR was performed on tumours from 25 patients from whom FF tissue was available. Relative mean *MGMT* expression expressed as -dCT was assessed by RT-Q-PCR using FF tissue available for 25 samples. Relative mean *MGMT* expression was significantly different for dSDH wtGIST (N=16) vs TK mutant GIST (N=9) (–2.194; SD 1.353 vs 0.33; SD 0.495, Mann-Whitney t-test (p<0.0001)). The relative mean *MGMT* expression for dSDH wtGIST caused by an *SDHC* epimutation (N=4) was −2.563; SD 2.063; vs −2.018; SD 1.119 for dSDH wtGIST caused by germline *SDHx* mutations (N=12) vs 0.330; SD 0.495 for TK mutant GIST (N=9). Analysis of variance (ANOVA;Kruskal-Wallis) p=0.0004. For samples where paired MGMT promoter methylation and MGMT Q-RT-PCR was available, no significant correlation was found on Pearson’s correlation.

### Correlation between *MGMT* methylation and clinicopathological parameters

Mean *MGMT* methylation levels were not significantly different in patients with metastatic disease (p=0.19) vs those with single vs multiple tumours (p=0.31), or those with a second synchronous primary tumour (p=0.32). No correlation was identified between mean methylation levels and the tumour proliferation index (p=0.48, R-score −0.105) or the tumour morphology (p=0.09).

### Correlation between *MGMT* methylation and *SDHx* subunit gene mutations in GIST and other *SDHx*-related tumours from the literature

To validate our findings, we assessed *MGMT* methylation status in previously published GIST datasets. Comparing dSDH (N=68) to pSDH (N=92) GIST samples from Killian *et al* 10, we identified a significantly (p=0.00057) higher mean *MGMT* methylation in dSDH GIST (figure 1A) (mean methylation in dSDH GIST 8.1%; STD 8.0% vs pSDH GIST 5.1%; STD 1.4%). To assess whether a mutation in a specific *SDHx* gene is a positive predictor of *MGMT* methylation, we analysed the expression of *MGMT* in a separate cohort of GISTs for which *SDHx* subunit mutational status is known (N=20)^18^ (figure 1B). We did not find significant differences in *MGMT* expression across tumours with different *SDHx* subunit gene mutations including cases with a confirmed *SDHC* epimutation (ANOVA p=0.43).

Finally, we assessed the impact of *SDHx* mutations on *MGMT* methylation in a tumour dataset of PPGL (online supplemental figure S2) (N=190).^16^ SDH-deficient PPGL demonstrated a significant (p<0.00001) increase in *MGMT* promoter methylation compared with pSDH PPGL (mean methylation in dSDH PPGL 15.5%; STD 11.3% vs pSDH PPGL 6.6%; STD 4.9%). We observed the previously reported significant increase in *MGMT* methylation for *SDHB* mutant PPGL compared with SDH preserved samples (ANOVA p<0.001, Tukey post hoc SDHB mut vs SDH WT q=0.001) (online supplemental figure S2).

## Discussion

The antitumour activity of TMZ has been demonstrated in a variety of MGMT-deficient tumours; including glioblastomas, gastroenteropancreatic neuroendocrine tumours and phaeochromocytomas/paragangliomas.^12 16 22^ The aim of the current study was to determine if *MGMT* methylation status could identify a subgroup of patients with GIST that may benefit most from TMZ therapy and to analyse potential correlations between molecular drivers of GIST, clinical and pathological parameters and *MGMT* methylation status.

We identified *MGMT* promoter hypermethylation in 8 patients and uniquely in SDH-deficient GIST (12.3% of study cohort and 22.4% of SDH-deficient GIST samples). dSDH wtGIST are a heterogeneous tumour subtype, typically presenting at a younger age and more frequently metastatic at presentation than TK-mutant GIST or SDH preserved wild-type GIST^17^ In contrast to TK mutant GIST, dSDH wtGIST can be indolent for long periods and median overall survival is often measured in many years,^5^ highlighting the need for well-tolerated therapies that are most likely to yield benefit for the individual patient. SDH deficiency detected by SDHB IHC, was the only predictor of *MGMT* hypermethylation in this large series of wild-type and TK mutant GIST.

Specific genotype–phenotype correlations have emerged for patients with germline *SDHx* mutations; *SDHB* variants are most commonly associated with RCC *SDHD* with head and neck paragangliomas and *SDHA* with GIST.^8 23 24^ The underlying shared mechanism of tumourigenesis for *SDHx* mutated tumours includes a complex interplay between succinate metabolism, metabolic reprogramming, redox imbalance and epigenetic regulation. The emerging genotype–phenotype correlations suggest that there may be tissue specific thresholds for altered succinate metabolism. It is assumed that the latter is further influenced by the specific *SDHx* subunit mutation and type of mutation, for example, missense versus truncating mutations.^25^ ^24–26^ Combining the results from our cohort with the data analysis of published datasets, no significant difference in mean *MGMT* methylation levels in SDH-deficient GIST with different underlying *SDHx* subunit gene mutations or *SDHC* epimutations, was identified. This is relevant as it suggests that *MGMT* promoter methylation analysis should be considered in all patients with wild-type GIST and in particular those with evidence of SDH deficiency on SDHB IHC, regardless of the underlying molecular driver.

**Figure 2:**
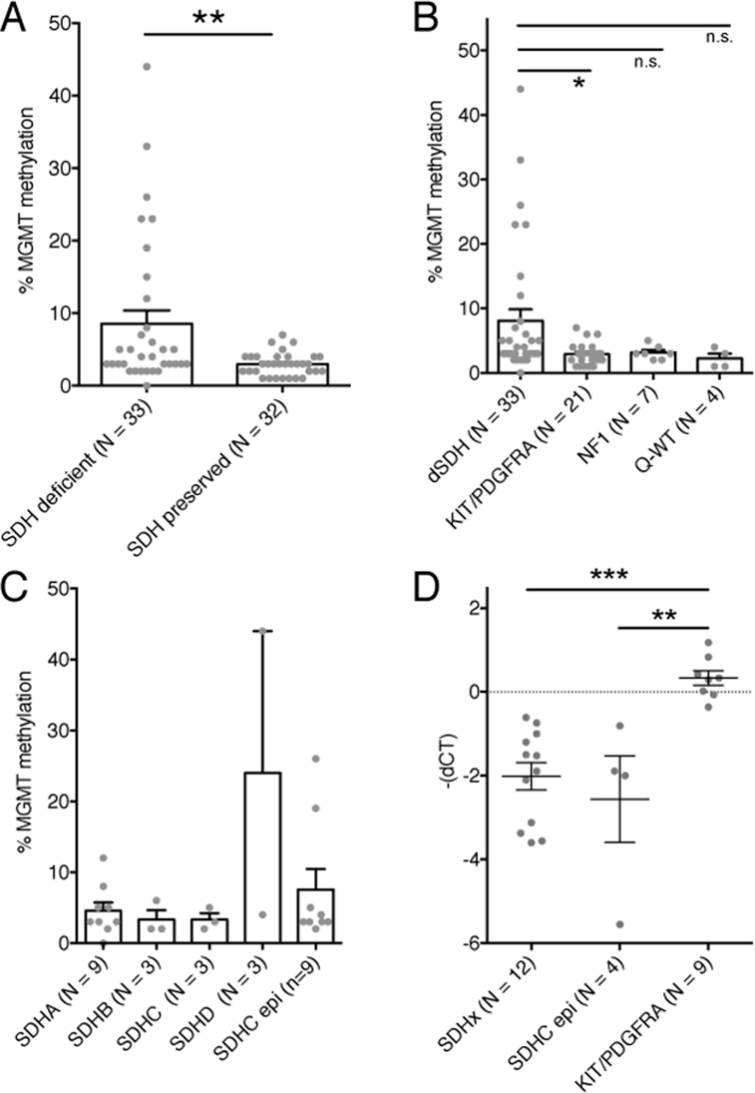
(A) Comparison of *MGMT* promoter methylation between dSDH and pSDH GIST; **p=0.0063 (Mann-Whittney t-test). (B) Comparison of *MGMT* promoter methylation between dSDH, KIT/PDGFRA mutated, NF1 associated and Q-WT GIST p=0.0449 one-way ANOVA (Kruskal-Wallis); *p=0.0134 (Mann-Whitney-test). (C) MGMT methylation in dSDH GIST groups. (D) MGMT-dCT values for expression in SDHx, SDHCepi and KIT/PDGFRA mutated GIST p=0.0004. One-way ANOVA (Kruskal-Wallis); **p=0.004, ***<0.0001 (Mann-Whitney-test). GIST, gastrointestinal stromal tumour.

In this study, we employed a pyrosequencing-based analysis of CpG’s 1–8 in the promoter region of the *MGMT* gene using bisulfite converted DNA from FFPE tumour samples, adopting a protocol commonly used in routine clinical practice for glioma samples. *MGMT* promoter hypermethylation correlated with reduced *MGMT* expression levels in dSDH GIST compared with pSDH wtGIST or TK mutant GIST (figure 2D). Pyrosequencing was favoured over methylation arrays for this study because of the wider availability of FFPE embedded tumour samples and for cost effectiveness. In the UK, the national genomic test directory, recommends methylation analysis using methylation arrays and targeted testing for example, *MGMT* or *MLH1* for a number of cancers including CNS tumours and other solid organ tumours (https://www.england.nhs.uk/publication/national-genomic-test-directories). At present, *MGMT* methylation analysis is not recommended for wtGIST as part of the national test directory but data from this study and others suggests that *MGMT* methylation analysis should be considered for patients with wild-type GIST and in particular those with evidence of SDH deficiency on SDHB IHC in order to identify patients who may benefit from TMZ therapy.

*MGMT* methylation status has been demonstrated to be an independent predictor of overall survival for patients with high-grade gliomas, irrespective of the treatment assignment.^12^ Interestingly, Lou *et al*’s data suggest MGMT promoter hypermethylation to be an independent favourable prognostic factor for overall and disease-free survival.^15^ The upcoming data from the clinical trial (*NCT03556384)* should inform the therapeutic benefit of TMZ for patients with inoperable dSDH GIST. However, independent of its predictive significance from a therapeutic standpoint, assessing *MGMT* methylation status may also provide prognostic information for patients with wtGIST, analogous to in high-grade gliomas^12^

In summary, our study of GIST found that *MGMT* promoter methylation is a recurrent epimutation exclusive to dSDH wtGIST (8 out of 33 dSDH GIST showed MGMT promoter hypermethylation, 22.4%). We found that there was no correlation between the underlying *SDHx* mutation or *SDHC* epimutation and the level of *MGMT* methylation, neither in our cohort of patients nor on secondary analysis of publicised datasets of GIST and PPGL. We did not identify additional clinical or pathological predictors for *MGMT* promoter methylation. *MGMT* promoter methylation analysis may be an important predictor of response to TMZ for patients with wtGIST and data from this study supports the routine utility of *MGMT* methylation analysis for patients with dSDH wtGIST in clinical practice.

## Data Availability

All data relevant to the study are included in the article or uploaded as online supplemental information. Not applicable.
