# Supplementary material for: Preferential MGMT hypermethylation in SDH-deficient wild-type GIST
Source: J Clin Pathol. 2022 Oct 5;77(1):34–9. doi: 10.1136/jcp-2022-208462 (PMC10804026; doi:10.1136/jcp-2022-208462)
Supplement: Supplementary data [file jcp-2022-208462supp001.pdf]

S - Material and Methods

v) DNA Extraction

DNA was extracted from either 2 mm punches taken from tissue in paraffin blocks with an Integra miltex biopsy punch (Integra, 33-31-P/25), marked areas from unstained paraffin sections mounted on glass slides (USS) or from fresh frozen tissue. Tissue from paraffin punches was deparaffinized by immersion in 100% xylene twice for 30 minutes and in 100% ethanol twice for 30 min, and air dried. The tissue punches were then incubated 1- 3 days at 56°C in 50-200 μl (volume depending on tissue block size) of proteinase K digestion buffer (PKDB) composed with 1x PCR buffer II (Applied Biosystems 4486220), 0.05% NP-40 and 200μg/ml proteinase K (Qiagen 19133). After incubation the proteinase was heat inactivated for 10 min at 96°C. The sample was let to cool to room temperature and then centrifuged for 1 min at 13.000 rpm. Tissue sections on USS were deparaffinized on slides, aligned to a corresponding slide stained with hematoxylin and eosin to demarcate tumour and normal adjacent regions and then scraped off into 30-100 μl PKDB buffer and treated the same as above. Fresh frozen tissue was added directly from frozen to PKDB buffer and processed similarly.

vi) RNA Extraction from Fresh Frozen Tissue

RNA from fresh frozen (FF) tissue was isolated with the Direct-zol RNA miniprep Kit (R2050, Zymo Research). A 25-50 mg block of FF tissue was transferred to a 50 ml Corex centrifuge tube with 600 μl of Biorad PureZol RNA Isolation reagent (Cat#732-6890) and homogenised with a small homogeniser tip for 3-4 x 20 sec burst with intermittent 30 sec cool down periods on ice. The tube was centrifuged for 5 min at 4°C at 3000 rpm and the supernatant transferred to a tube containing 600 μl 100% ethanol and mixed by inversion. The mix was transferred to a Zymo-Spin IIC Column and centrifuged for 1 min at 8000 rpm. After one wash with 400 μl RNA wash buffer a DNAseI treatment was performed (5 μl of DNAseI +75 μl of DNase buffer/column) for 15 min at RT. One wash with 400 μl RNA pre-wash buffer, one wash with 700 μl RNA wash buffer with a 2 min centrifugation was performed before elution with 100μl RNAse free water. Eluted RNA was

quantified by Nanodrop ND-1000 Spectrophotometer and Quality assessed with RNA Tapescreen(™) on a TapeStation(™). For RNA extraction FFPE tissue scraped from slides (USS) or punched from paraffin blocks (as described above) were processed with the RNAstorm kit from CellDataSci (CD501) according to the manufacturer's protocol.

vii) Bislufite conversion

200 ng DNA was bisulfite modified with either the Qiagen Epitect Bisulfite kit (Cat 59104) or the Zymo Research EZ DNA Methylation kit (D5001) according to the manufacturers' instructions. Complete bisulfite modification was monitored by an internal bisulfite control position in the pyrosequencing assays for MGMT and SDHC.

viii) Analysis of MGMT and SDHC promoter methylation

MGMT: A 124 bp sized PCR amplicon located in the promoter region of the MGMT gene was amplified from 1-25 ng bisulfite converted DNA with 375 nM forward primer, with a 20-mer 5' M13 overhang to facilitate post PCR sequencing, (TGTAAAACGACGGCCAGTTTATAGTTTYGGATATGTTGGGATAG) and 187.5nM of biotinylated reverse primer ([btn]-TCCCAAACACTCACCAAATC) with the Qiagen PyroMark kit (Qiagen 978703).

SDHC: For methylation analysis of the SDHC gene a 134 bp PCR amplicon located in the promoter region of the SDHC gene was amplified from 2-25 ng of bisulfite converted DNA with 375 nM forward primer, with a 20-mer 5' M13 overhang to facilitate post PCR sequencing, (TGTAAAACGACGGCCAGTTTATAGGAGAAGTTTTAGAGTTTTTTAAAGAG) and 250nM of biotinylated reverse primer ([btn]-AAAATAACRCCAAACRACCCC). The PCR conditions were 7 min at 95°C, followed by 20 sec at 95°C, 30 sec at 53°C, and 20 sec at 72°C for 40 cycles, and an end incubation at 72°C for 5 min. The resulting PCR amplicon was quality assessed for purity and

yield on a 2% agarose gel. For MGMT, a nested sequencing primer (GTTTTTAGAACGTTTTGYGTTT) was used to analyse 4 CpGs in 10 μl of the PCR sample on the Qiagen Q24 pyrosequencer (sequence to analyse: YGAYGTTYGTAGGTTTTYGT). For SDHC a nested sequencing primer (GTTATATGATATTTTTAATTT) was used to analyse six CpGs (sequence to analyse: YGATTTTTAGTYGGYGYGTTTTYGTTTTYGGGT). Fully methylated and unmethylated human control DNA that had been treated with bisulfite were used as controls on each pyrosequencing run. Pyrosequencing results were confirmed in a subset of cases by Sanger sequencing with the M13 forward primer recognising the M13 extension of the forward oligos for both the MGMT and the SDHC PCR amplicon.

ix) MGMT expression analysis with quantitative RT-PCR

RNA (125-250 ng) was transcribed into cDNA with random hexamers employing the High Capacity cDNA Reverse Transcription Kit from Applied Biosystems (ref 4368814) according to manufacturer's instructions. cDNA was diluted tenfold after synthesis and 1% was used in each RT-PCR well. Relative MGMT expression was analyzed according to (Uno et al 2011) (16) with SYBR Green using the PowerUp SYBR Green Master Mix (Applied Biosystems, ref 01061935), with MGMT oligo's; forward 5'-GCTGAATGCCTATTTCCACCA-3'/reverse 5'-CACAACCTTCAGCAGCTTCCA-3'; normalised to the average Ct value of three internal reference genes; hypoxanthine guanine phosphoribosyltransferase (HPRT1); forward 5'- GAAAAGGACCCCACGAAGTGT -3'/ reverse 5'- AGTCAAGGGCATATCCTACAACA -3', beta-glucuronidase (GUSB); forward 5'- AAAATACGTGGTTGGAGAGCTCATT -3'/ reverse 5'- CCGAGTGAAGATCCCCTTTTTA -3'and TATA-box binding protein (TBP); forward 5'- AGGATAAGAGAGCCACGAACCA -3'/ reverse 5'- CTTGCTGCCAGTCTGGACTGT -3'. The delta Ct was calculated by subtracting the mean of triplicate Ct values for MGMT with the mean of the triplicate Ct values of all 3 reference genes.

MGMT promoter hypermethylation was defined as a mean MGMT methylation across CpG islands 1-8 of > 10% for this study.

Clinical germline DNA sequencing

DNA was extracted from peripheral blood samples according to standard protocols. Next generation sequencing of a clinical gene panel including; *SDHA*, *SDHB*, *SDHC*, *SDHD*, *KIT, PDGFRA* and *NF1* was performed by the laboratory staff at Cambridge University Hospital NHS Foundation Trust or Birmingham Women's and Children's Hospital NHS Trust using the TrusightOne or Trusight Cancer sequencing panels (Illumina Inc., UK). An average coverage depth of >20 fold was achieved for 98% of the regions sequenced. All detected variants were confirmed by Sanger sequencing. Whole exon deletions and duplications and large rearrangements are not detected using this method and multiple ligation probe analysis (MLPA) was performed for *SDHB*, *SDHC* and *SDHD*.

xii) SDHB Immunohistochemistry

SDHB immunohistochemistry (IHC) was performed on 4 µm sections of FFPE tissue using a commercially available SDHB polyclonal rabbit antibody (Sigma-Aldrich HPA002868). Heat-induced epitope retrieval was carried out using a Leica heat retrieval solution (HPA002867, Sigma Aldrich, UK). SDHB deficiency was defined by a loss or abnormal IHC staining pattern for SDHB in the tumour cells when compared to the staining pattern in tumour vasculature cells.
