# Supplementary material for: Preferential MGMT hypermethylation in SDH-deficient wild-type GIST
Source: J Clin Pathol. 2022 Oct 5;77(1):34–9. doi: 10.1136/jcp-2022-208462 (PMC10804026; doi:10.1136/jcp-2022-208462)
Supplement: Supplementary data [file jcp-2022-208462supp002.pdf]

Table S1: Mean methylation for TK mutant GIST and pSDH wtGIST

Tumour ID	KIT/PDGFRA mutation status	SDH status	Germline gene mutation	Mean MGMT methylation% CpG 1-8
G0054	KIT	SDHp	NA	3%
G0064	KIT	SDHp	KIT c.1924A>G (p.K642E) homozygous ;c.2466T>A, (p.N822K)	1%
G0072	KIT	SDHp	KIT c.1504_1509 dupGCCTAT p.(Ala502_Tyr503dup)	3%
G0074	KIT	SDHp	KIT c.1701_1728del p.(Asn567_Leu576delinsLysGlu)	4%
G0075	KIT	SDHp	KIT c.1509_1510insGCCTAT p.(Ser501_Ala502insAlaTyr)	7%
G0101	KIT	SDHp	KIT c.1669T>C p.(Trp557Arg)	1%
G0103	KIT	SDHp	KIT p.(Leu576Pro)	3%
G0104	KIT	SDHp	KIT c.1669_1674del p.(Trp557_Lys558del)	4%
G0105	KIT	SDHp	KIT p.(Trp557Arg)	6%
G0106	KIT	SDHp	KIT E11 mutation	3%
G0112	KIT	SDHp	KIT c.1668_1724del p.(Trp557_Thr574del) c.2460T>G p.(Asp820Glu)	1%
G0113	KIT	SDHp	KIT c.1738_1739insTAGACCCAACACAACTTCCTTATGATC p.(Ile571_Asp572insAspProThrGlnLeuProTyrAspLeu)	1%
G0116	KIT	SDHp	KIT c.1679T>A p.(Val560Asp)	3%
G0145	KIT	SDHp	KIT E11 deletion	2%
G0146	KIT	SDHp	KIT c.1668_16679delGTGGAAGGTTGTinsTTCCAC p.(Glu556_Val560_insHisSerThr)	1%
G0264a2	KIT	SDHp	KIT p.(Trp557Gly) SDHA c1A>C, (p.MET1?) (p.MET1?)	2%
G0264b1	KIT	SDHp	KIT p.(Val559Asp)	2%
G0032	NF1	SDHp	NF1 c.7706dupA p.(His2569GlnfsTer6)	3%
G0039	NF1	SDHp	NA	3%
G0041	NF1	SDHp	NA	2%
G0044	NF1	SDHp	NA	3%
G0048b	NF1	SDHp	NA	2%
G0083	NF1	SDHp	NA	5%
G0109	NF1	SDHp	NA	4%
G0060	PDGFRA	SDHp	PDGFRA c.2525A>T p.(Asp842Val)	2%
G0061	PDGFRA	SDHp	PDGFRA c.2526_2537delCATCATGCATGA p.(Asp842_His845del)	6%
G0102	PDGFRA	SDHp	PDGFRA c.1977C>G p.(Asn659Lys)	4%
G0149	PDGFRA	SDHp	PDGFRA p.(Val561Asp)	2%
G0046	Quad.	SDHp	NA	3%
G0048a	Quad.	SDHp	NA	3%
G0052	Quad.	SDHp	NA	4%
G0056	Quad.	SDHp	NA	1%
G0110	Quad.	SDHp	NA	1%
G0138	NA	SDHp	NA	4%
G0139	NA	SDHp	NA	4%

BMJ Publishing Group Limited (BMJ) disclaims all liability and responsibility arising from any reliance placed on this supplemental material which has been supplied by the author(s)

Table S2: Mean methylation in tumour and adjacent tissue

Sample ID	KIT/PDGFRA mutation status	SDH status	Germline gene mutation	Mean MGMT methylation % of tumour CpG 1-8	Mean MGMT methylation %of adjacent normal CpG 1-8
G0002N	-	SDHp	Adjacent normal of SDHd	2%	4%
G0003N	-	SDHp	Adjacent normal of SDHd	23%	4%
G0013N	-	SDHp	Adjacent normal of SDHd	5%	2%
G0017N	-	SDHp	Adjacent normal of SDHd	3%	5%
G0018N	-	SDHp	Adjacent normal of SDHd	2%	3%
G0019N	-	SDHp	Adjacent normal of SDHd	5%	4%
G0024N	-	SDHp	Adjacent normal of SDHd	7%	8%
G0044N	-	SDHp	Adjacent normal of NF1	3%	10%
G0053N	-	SDHp	Adjacent normal of SDHd	4%	1%
G0054N	-	SDHp	Adjacent normal of KIT	3%	1%
G0074N	-	SDHp	Adjacent normal of KIT	4%	4%
G0081N	-	SDHp	Adjacent normal of SDHd	2%	2%
G0082N	-	SDHp	Adjacent normal of SDHd	19%	2%
G0083N	-	SDHp	Adjacent normal of NF1	5%	11%
G0085N	-	SDHp	Adjacent normal of SDHd	3%	4%
G0086N	-	SDHp	Adjacent normal of SDHd	3%	3%
G0101N	-	SDHp	Adjacent normal of KIT	1%	4%
G0138N	-	SDHp	Adjacent normal	4%	6%
G0150N	-	SDHp	Adjacent normal of SDHd	3%	2%
G0151N	-	SDHp	Adjacent normal of SDHd	26%	4%

Figure S1

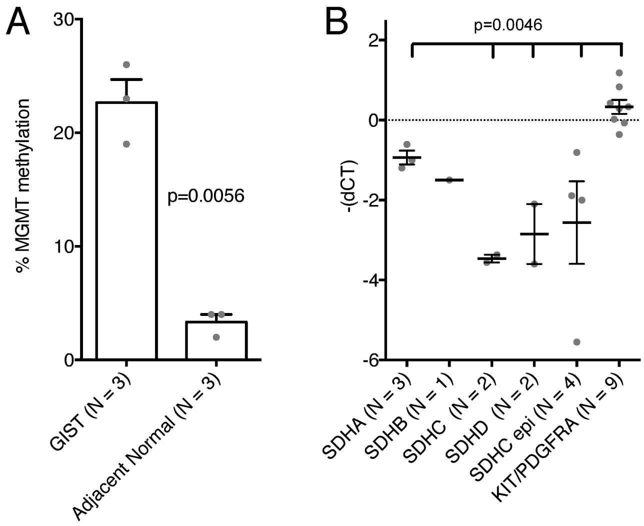

A) MGMT promoter methylation in GIST in comparison to adjacent normal tissue where elevated methylation was observed in the tumour (N = 3; p=0.0056). B) Detailed listing of -dCT values in GIST (q-RT-PCR). Kruskall-Wallis one way ANOVA for MGMT expression (SDHB excluded): p=0.0046.

Figure S2 (Related to Figure 2). MGMT promoter methylation for patients WT and mutant for SDH.

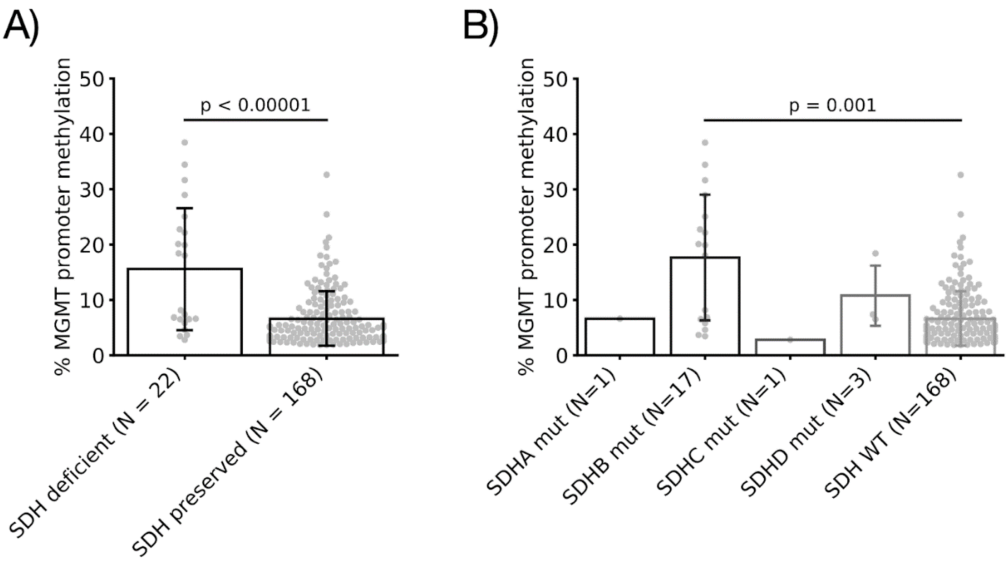

A) PPGL patients deficient for SDH show an increased MGMT promoter methylation; p represents student's t-test. B) PPGL patients published by Hadoux et al show no differences between SDH mutant patients. Patients mutant for SDHB show an increase in MGMT promoter methylation compared to SDH WT. ANOVA $p < 0.001$; p represents multiple test corrected Tukey post-hoc.
